# Supplementary material for: Chromatin landscape at cis-regulatory elements orchestrates cell fate decisions in early embryogenesis
Source: Nat Commun. 2025 Mar 27;16:3007. doi: 10.1038/s41467-025-57719-4 (PMC11950382; doi:10.1038/s41467-025-57719-4)
Supplement: Supplementary file 1 — Supplementary Information [file 41467_2025_57719_MOESM1_ESM.pdf]

Supplementary Information

Chromatin landscape at *cis*-regulatory elements orchestrates cell fate decisions in early embryogenesis

Author list: Francesco Cardamone[1,2,3]*, Annamaria Piva[4]*, Eva Löser[1], Bastian Eichenberger[4], Mari Carmen Romero-Mulero[1,2], Fides Zenk[5], Emily J. Shields[6,7,8], Nina Cabezas-Wallscheid[1,9,10], Roberto Bonasio[6,7], Guido Tiana[11], Yinxiu Zhan[4#], Nicola Iovino[1#]

Affiliations:

[1] Max Planck Institute of Immunobiology and Epigenetics, 79108, Freiburg, Germany.
[2] Faculty of Biology, University of Freiburg, 79104, Freiburg, Germany.
[3] International Max Planck Research School of Immunobiology, Epigenetics and Metabolism (IMPRS-IEM), 79108 Freiburg, Germany.
[4] Department of Experimental Oncology, European Institute of Oncology, IRCCS, Milan, Italy.
[5] Epigenomics of Neurodevelopment, Brain Mind Institute, School of Life Sciences, EPFL – Ecole Polytechnique Federal Lusanne, Station 19, 1015 Ecublens, Switzerland.
[6] Epigenetics Institute, Department of Cell and Developmental Biology, University of Pennsylvania Perelman School of Medicine, Philadelphia, PA 19104, USA.
[7] Department of Cell and Developmental Biology, University of Pennsylvania Perelman School of Medicine, Philadelphia, PA,19104, USA.
[8] Department of Urology and Institute of Neuropathology, Medical Center–University of Freiburg, 79106 Freiburg, Germany.
[9]Laboratory of Stem Cell Biology and Ageing, Department of Health Sciences and Technology, Swiss Federal Institute of Technology (ETH Zürich), Zürich, Switzerland.
[10]Centre for Integrative Biological Signalling Studies (CIBSS), Freiburg, Germany.
[11] Università degli Studi di Milano and INFN Milan Italy.

*These authors contributed equally.

# Corresponding authors: yinxiu.zhan@ieo.it, iovino@ie-freiburg.mgp.de

Table of contents

Supplementary Figures 1-10.

Description of Supplementary Data 1-9.

Supplementary Figure 1

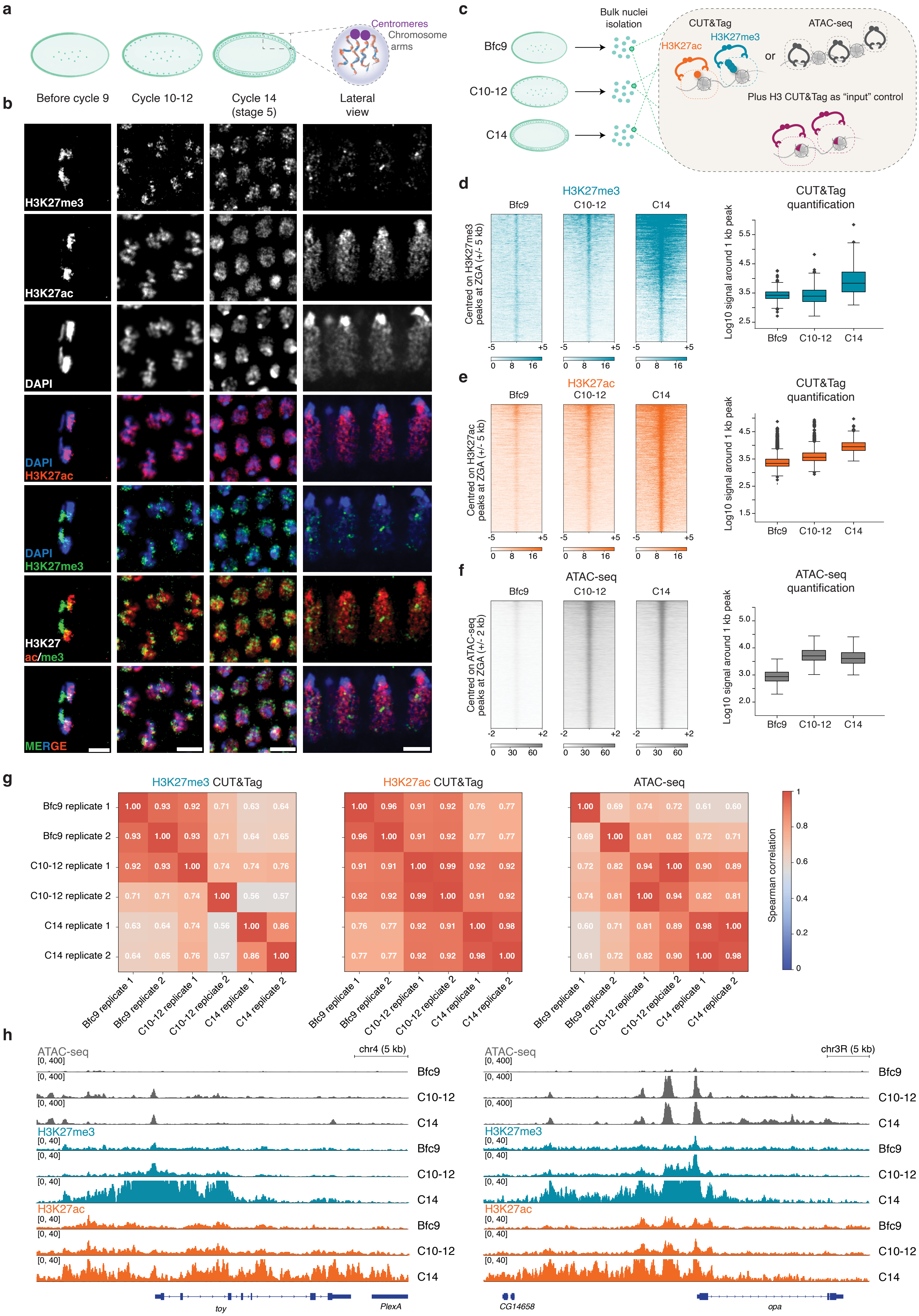

Supplementary Fig. 1: Comprehensive overview of the chromatin landscape during early *Drosophila* development.

a, Representation of early *Drosophila* development from before cycle 9 to cycle 14 (ZGA). Cartoon of lateral view of the ZGA nucleus in the RABL configuration: centromeres are highlighted in purple, chromosome arms in grey, H3K27me3 in blue, H3K27ac in orange.

b, Immunofluorescence staining of H3K27me3, H3K27ac and DAPI across three developmental time points. Representative image from three biological replicates. Scale bar 5 µm.

c, Experimental design. Early embryos are hand-selected before cycle 9, at cycle 10-12 or at cycle 14 (ZGA) based on their morphology. Nuclei are isolated and processed for CUT&Tag or ATAC-seq. Each experiment is normalized against its H3 CUT&Tag (see Methods). Bfc9, before cycle 9. C10-12, cycle 10-12. C14, cycle 14.

d, Left, Heat maps of H3K27me3 CUT&Tag normalized signal was centred on +/- 5kb H3K27me3 peaks at from before cycle 9 to cycle 14. Right, Boxplot quantifications of H3K27me3 signal within 1kb around peaks at three developmental time points. Boxes center refers to median, lower and upper quartiles (Q1 and Q3, respectively). Whiskers, 1.5 × IQR below Q1 and above Q3. Points denote outliers.

e, Left, Heat maps of H3K27ac CUT&Tag normalized signal (see Methods) was centred on +/- 5kb H3K27ac peaks at from before cycle 9 to cycle 14. Right, Boxes center refers to median, lower and upper quartiles (Q1 and Q3, respectively). Whiskers, 1.5 × IQR below Q1 and above Q3. Points denote outliers.

f, Left, Heat maps of ATAC-seq normalized signal (see Methods) was centred on +/-2kb ATAC-seq peaks at ZGA. Right, Boxplot quantifications of ATAC-seq signal within 1kb around peaks at three developmental time points. Boxes center refers to median, lower and upper quartiles (Q1 and Q3, respectively). Whiskers, 1.5 × IQR below Q1 and above Q3.

g, Spearman correlation coefficient heat maps across replicates for H3K27me3, H3K27ac CUT&Tag and ATAC-seq. Bfc9, before cycle 9; C10-12, cycle 10-12; C14, cycle 14.

h, Genome browser snapshot of two loci at three developmental time points. ATAC-seq tracks are displayed in grey, H3K27me3 tracks in blue, H3K27ac in orange. Each track is the resultant of pooling of two biological replicates. Bfc9, before cycle 9. C10-12, cycle 10-12. C14, cycle 14.

Supplementary Figure 2

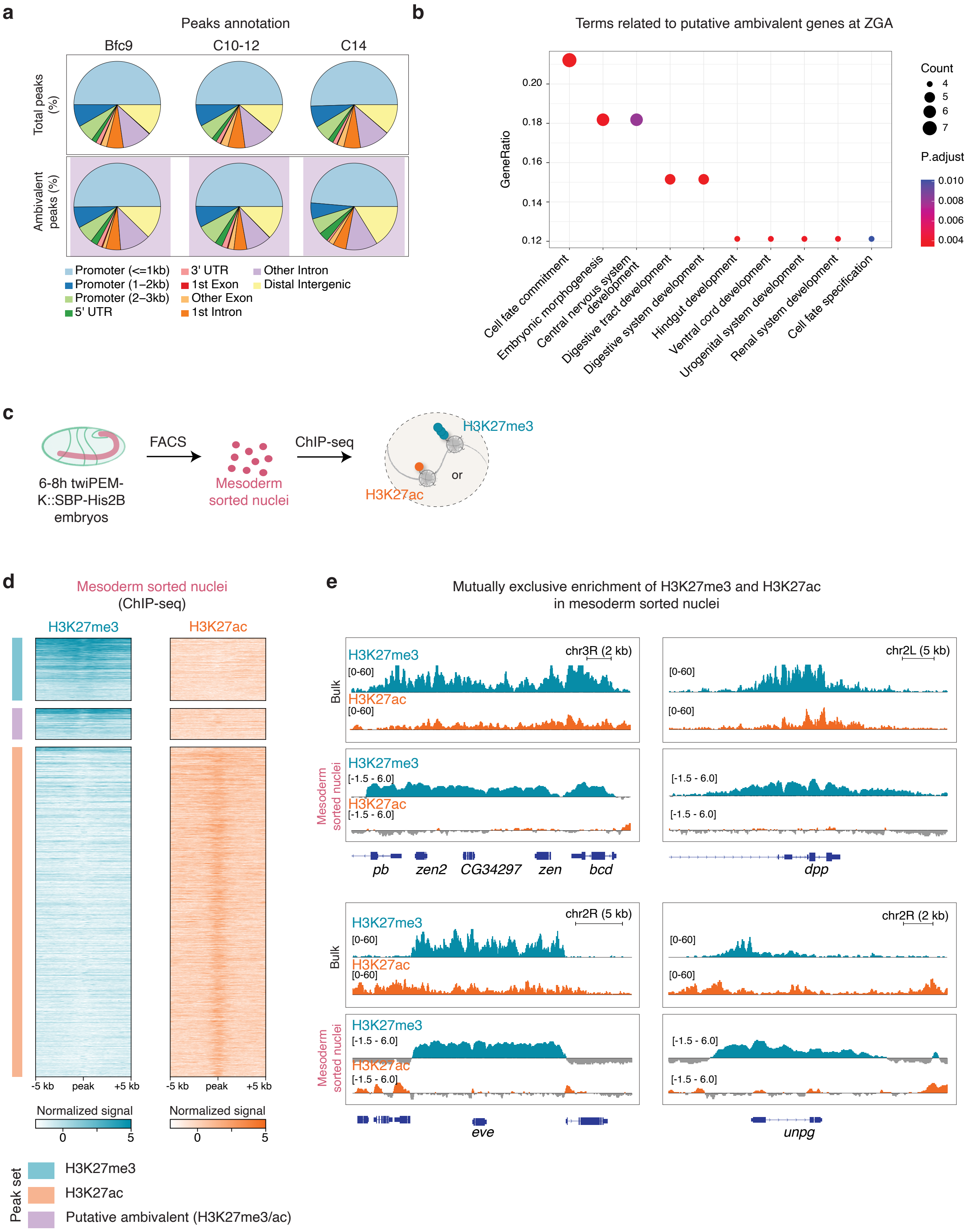

Supplementary Fig. 2: Ambivalent domains are not detected in mesoderm sorted nuclei.
a, Top, pie charts representing positional information of H3K27me3 or H3K27ac total peaks at three developmental time points. Bottom, pie charts representing positional information of ambivalent peaks at cycle 14.
b, Gene ontology of genes associated with ambivalent specific peaks at cycle 14 (ZGA). Source data are provided as Source Data file.
c, Experimental design. Mesoderm nuclei are FACS-sorted and isolated and ChIP-seq for H3K27me3 or H3K27ac is performed as described in[44].
d, Heat maps of mesoderm specific H3K27me3 (this study) or H3K27ac ChIP-seq[44]. Blue cluster represents H3K27me3 specific peaks, purple cluster represents ambivalent peaks and orange cluster represents H3K27ac specific peaks.
e, Genome browser snapshots of putative ambivalent cluster associated genes for H3K27me3 or H3K27ac bulk CUT&Tag or mesoderm specific ChIP-seq.

Supplementary Figure 3

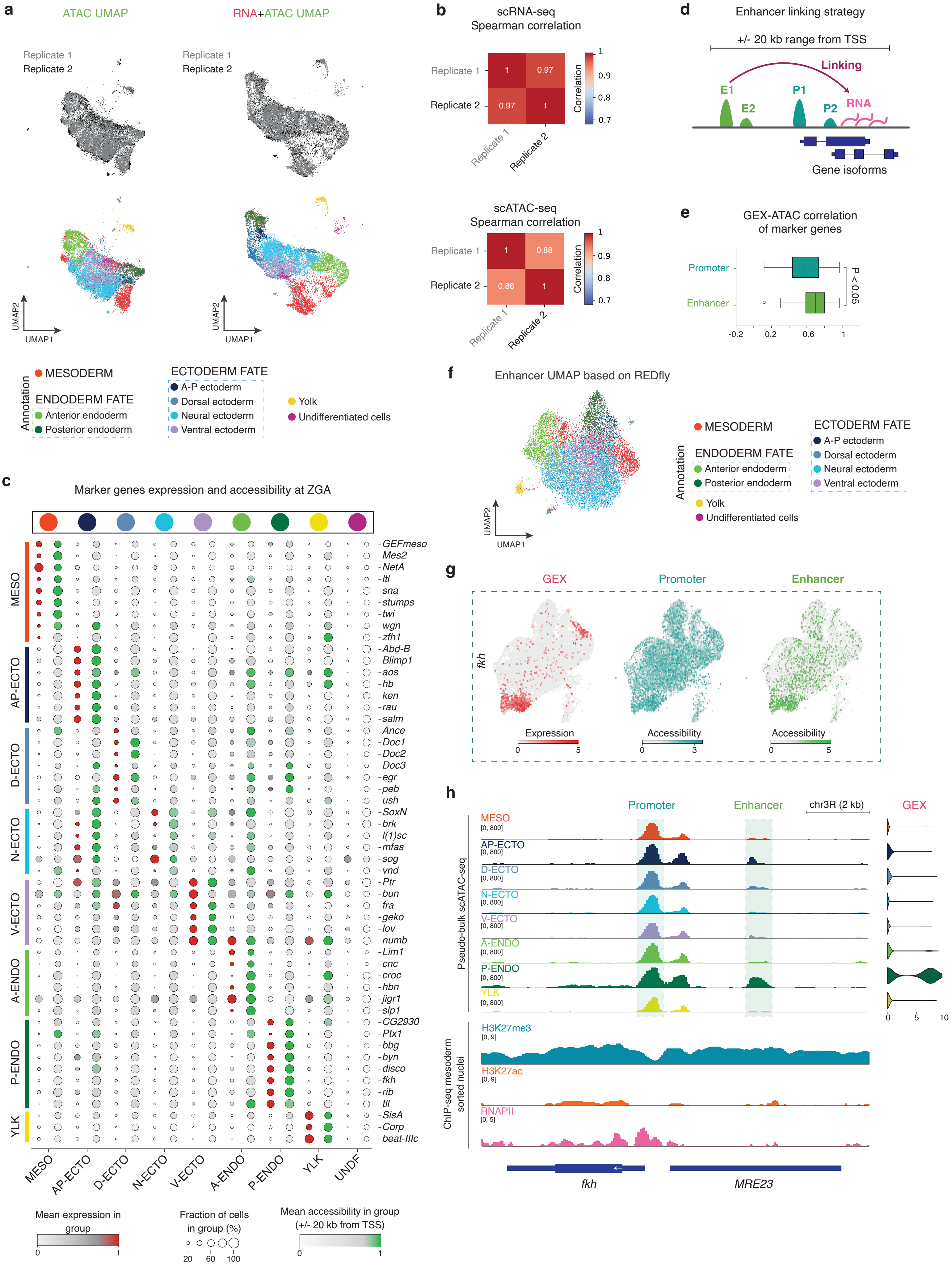

Supplementary Fig. 3: Single-cell multiomic profiling of the wild-type ZGA embryo.
a, UMAP embedding of 10x Multiome scATAC-seq (ATAC) or scRNA-seq + scATAC-seq (RNA+ATAC) from same nuclei. Cells from different biological replicates are indicated. Cluster identities were assigned by expression of marker genes.
b, Spearman correlation coefficient heat maps across biological replicates of the scRNA-seq and scATAC-seq data from wild-type ZGA embryos.
c, Dot plot showing gene expression and chromatin accessibility signal in a range window of +/- 20kb from TSS of selected marker genes per germ layer.
d, Schematic representation of the linking strategy adopted to assign enhancers to germ layer specific genes. Linking between gene expression and accessibility of peaks across +/- 20 kb from TSS is calculated (see Methods). The highest score linked peak is then considered the putative enhancer. The most accessible promoter is selected to study only transcribed isoforms at ZGA.
e, Distribution of spearman correlation between 10x Multiome scRNA-seq (GEX) and scATAC-seq (ATAC) of germ layer marker genes at their most accessible promoter peak or at their highest score linked peak (enhancer). Boxes, lower and upper quartiles (Q1 and Q3, respectively). Whiskers, 1.5 × IQR below Q1 and above Q3. Outliers are shown. Two-sided Mann-Whitney U test. *P*= 0.0204.
f, UMAP embedding of chromatin accessibility based on overlapping peaks with cis-regulatory modules (CRMs) from REDfly database, excluding promoter peaks.
g, scRNA-seq (GEX) or scATAC-seq of most accessible promoter peak or highest score linked peak (enhancer) of *fkh* (posterior endoderm marker gene).
h, Genome browser snapshot of *fkh* locus. Top, aggregated scATAC-seq reads of each germ layer and violin plot of the respective *fkh* gene expression (GEX). Promoter is highlighted by light blue dashed box, enhancer is highlighted by green dashed box. Bottom, ChIP-seq signal of H3K27me3 (this study), H3K27ac and RNA polymerase II (RNAPII) from mesoderm sorted nuclei[44].

Supplementary Figure 4

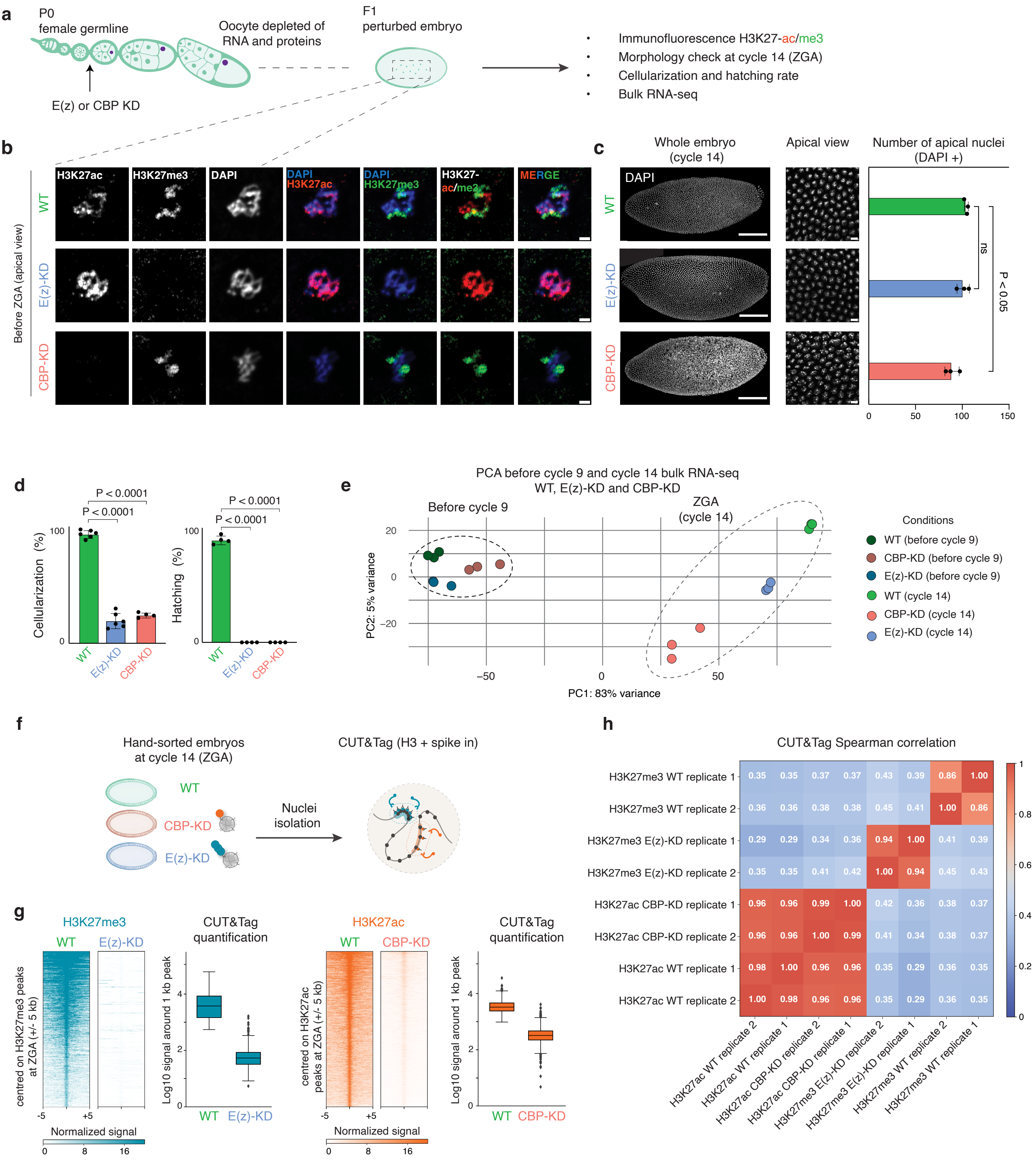

Supplementary Fig. 4: Phenotypic and molecular characterization of chromatin factors depleted embryos during early embryogenesis.

a, Schematic illustration of germline RNAi induced knock-down. The arrow indicates the knockdown activation in the ovariole. The purple oocyte is devoid of E(z) or CBP, resulting in F1 embryos devoid of the respective enzyme/histone mark. Embryos are subsequently characterized. Adapted from an image created in BioRender. Cardamone, F. (2025) https://BioRender.com/s77z537.

b, Immunofluorescence staining of pre-ZGA embryos for H3K27ac and H3K27me3 in both wild-type, CBP-KD and E(z)-KD. Representative image from three biological replicates per each condition. Scale bar, 2 µm.

c, DAPI staining of representative embryos at ZGA (cycle 14) in control and upon KD. Scale bar, 100 µm. A representative apical section from three embryos per each condition is showed. Scale bar, 5 µm. Quantification of nuclei number (DAPI +) per each condition is assessed from the apical area in three biological replicates. Ordinary one-way ANOVA. P= 0.0314. Data are presented as mean values ± SD. Source data are provided as Source Data file.

d, Phenotypic characterization of wild-type (WT), CBP-KD and E(z)-KD embryos. Percentage of embryos (top) reaching the ZGA stage or (bottom) completing embryogenesis (Hatching) Ordinary one-way ANOVA. $n = 600$ embryos for wild-type, $n = 400$ embryos for CBP-KD, $n = 600$ for E(z)-KD embryos. Data are presented as mean values ± SD. Source data are provided as Source Data file.

e, Principal component analysis (PCA) plot of total RNA-seq from before cycle 9 and cycle 14 embryos in control and KD conditions. Each dot represents a biological replicate. The dashed circles indicate the two time points. Source data are provided as Source Data file.

f, Experimental design. Wild-type (WT), E(z)-KD or CBP-KD embryos are hand-selected at cycle 14. Nuclei are isolated and processed for CUT&Tag. Each experiment is normalized (see Methods).

g, Heat maps of H3K27me3 or H3K27ac, CUT&Tag normalized signal was centred on +/- 5kb H3K27me3 or H3K27ac peaks at ZGA respectively in wild-type, E(z)-KD or CBP-KD. Box plots of H3K27me3 or H3K27ac peak signal within in +/- 1kb window. Boxes center refers to median, lower and upper quartiles (Q1 and Q3, respectively). Whiskers, 1.5 × IQR below Q1 and above Q3. Points denote outliers.

h, Spearman correlation coefficient heat maps across replicates and conditions for H3K27me3 and H3K27ac CUT&Tag.

Supplementary Figure 5

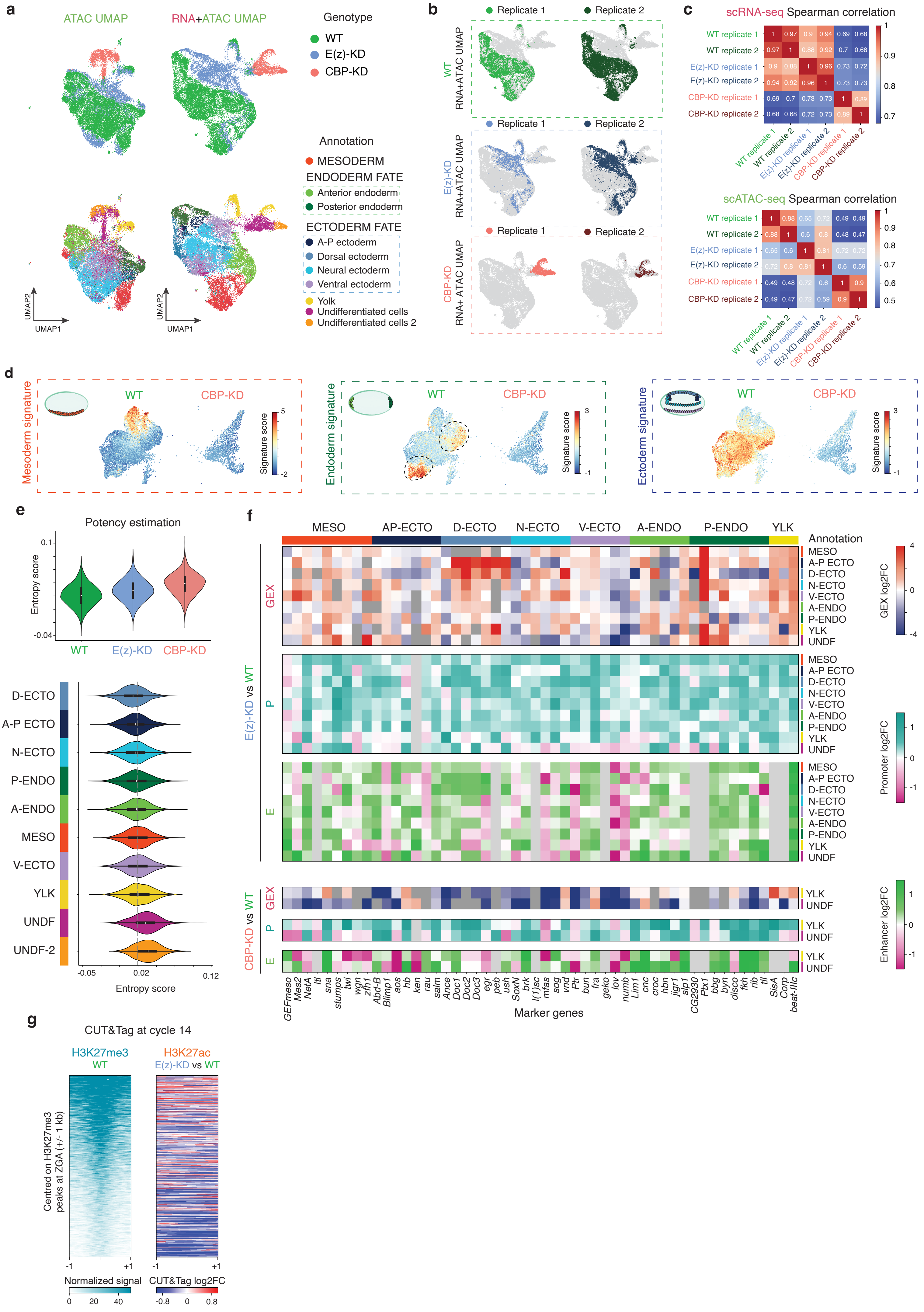

Supplementary Fig. 5: Single-cell multiomic profiling of chromatin factors depleted embryos.

a, UMAP embedding of 10x Multiome scATAC-seq (ATAC) or scRNA-seq + scATAC-seq (RNA+ATAC) from same nuclei. Cluster identities were assigned by genotype (top) or annotated germ layers based on expression of marker genes (bottom).

b, UMAP embedding of 10x Multiome scRNA-seq + scATAC-seq (RNA+ATAC) across biological replicates from wild-type and KD conditions. Cells from different biological replicates are indicated.

c, Spearman correlation coefficient heat maps across biological replicates of the scRNA-seq and scATAC-seq data from wild-type and KD conditions.

d, UMAP embedding with mesoderm, ectoderm and endoderm gene signature scoring in wild-type and CBP-KD. Color encodes signature enrichment.

e, Single-cell entropy score across different conditions (top) and germ layers (bottom). Higher entropy score delineates a higher pluripotency/uncertainty.

f, Differential gene expression and accessibility at promoter or enhancer of marker genes upon E(z)-KD or CBP-KD. The heat map shows ectopic expression and widespread increase of accessibility of marker genes upon loss of E(z), whereas complete shut-down of transcription without chromatin compaction is observed upon CBP-KD. Genes expressed in less than 1% of cells and missing peaks are represented with a grey square.

g, Heat maps of H3K27me3 in wild-type (WT) cycle 14 embryos and differential CUT&Tag enrichment between E(z)-KD and wild-type for H3K27ac at cycle 14. Sorting is based on H3K27me3 signal in wild-type embryos. The heat map was centred on +/- 1 kb H3K27me3 peaks at ZGA.

Supplementary Figure 6

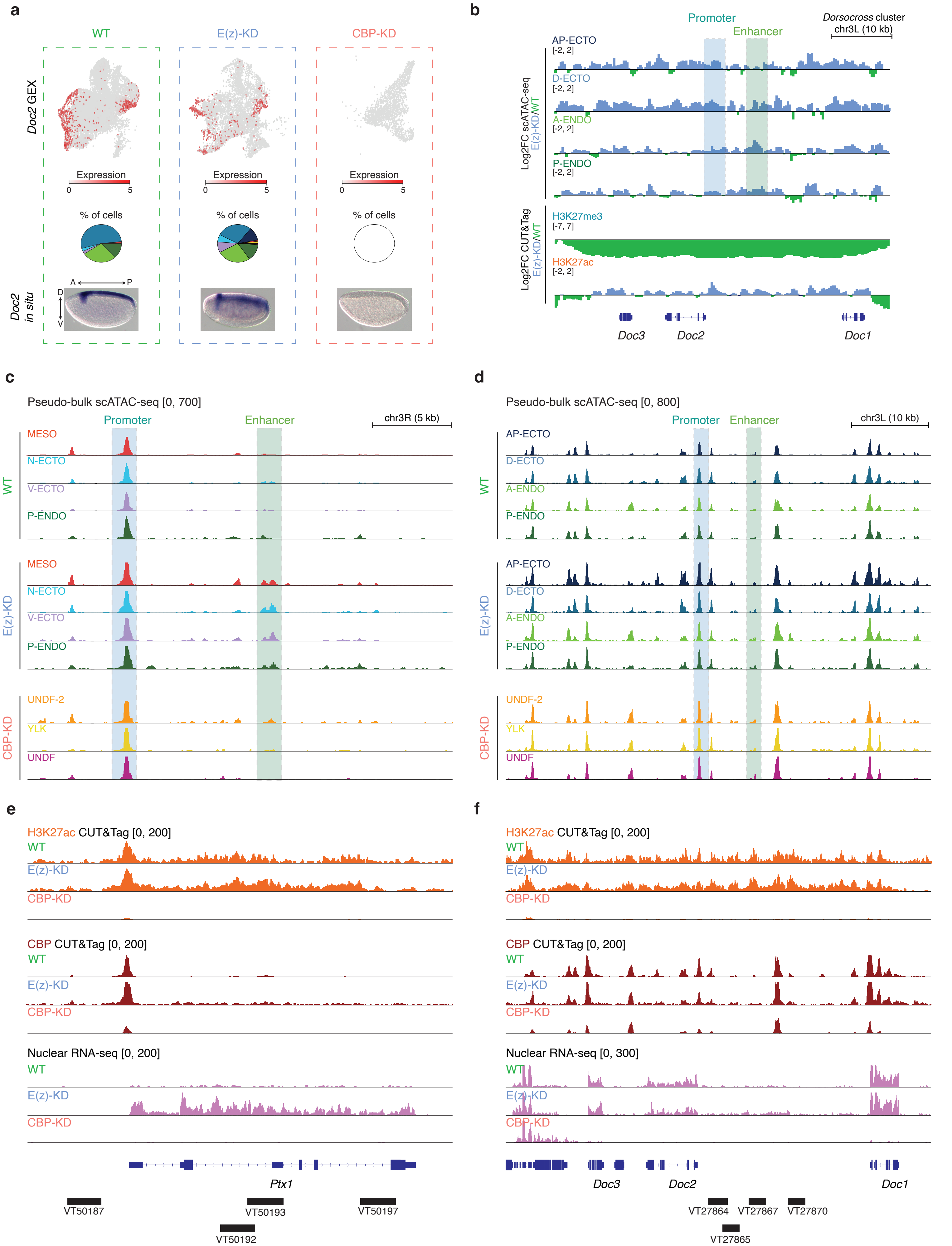

Supplementary Fig. 6: *Ptx1* and *Doc2* loci exemplify the epigenetic and transcriptomic changes upon perturbing chromatin factors.

a, Top, Gene expression (GEX) of *Doc2* (dorsal ectoderm marker gene) in wild-type, E(z)-KD and CBP-KD. Pie charts represent fraction of cells expressing the gene within each germ layer. Bottom, *in situ* hybridization of *Doc2* RNA in WT, E(z)-KD and CBP-KD validating the ectopic expression or transcriptional shutdown in perturbed embryos.

b, Genome browser snapshot of *Doc2* locus. Differential accessibility between E(z)-KD and wild-type cells in different germ layers. Promoter is highlighted with a blue dash box while enhancer is highlighted with a green dash box. Differential CUT&Tag signal between E(z)-KD and wild-type for H3K27me3 and H3K27ac.

c, d, Genome browser snapshot of *Ptx1* and *Doc2* loci. Aggregated scATAC-seq for germ layers in wild-type (WT), E(z)-KD and CBP-KD data. Promoter region is highlighted in blue, enhancer region is highlighted in green.

e, f, CUT&Tag signal for H3K27ac and CBP at *Ptx1* and *Doc2* loci in wild-type, E(z)-KD and CBP-KD. Nuclear RNA-seq signal in WT, E(z)-KD and CBP-KD. Refseq genes and regulatory elements from Fly Enhancers database (enhancers.starklab.org)[36]. Each regulatory element is displayed at cycle 14 (ZGA) and post ZGA.

Supplementary Figure 7

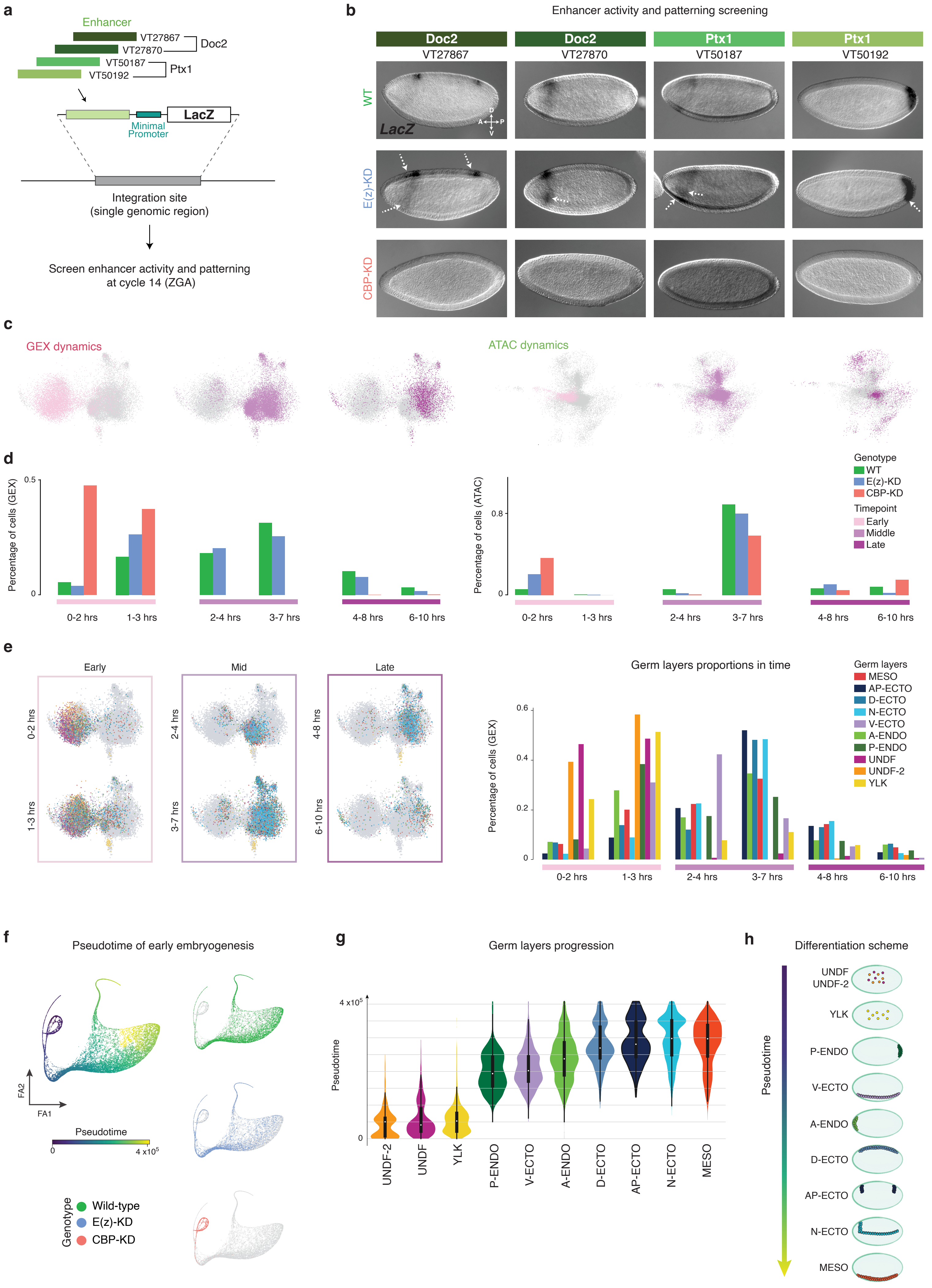

Supplementary Fig. 7: *In vivo* reporter assay of enhancer activity and patterning highlights aberrant phenotypes upon chromatin factors depletion.

a, Strategy for constructing transgenic lines to test selected enhancer activity and patterning for *Ptx1* and *Doc2* in wild-type and KD embryos. Candidate enhancers are inserted upstream of a transcriptional reporter and integrated in a single genomic region of the fly genome.

b, *In situ* hybridization for LacZ RNA by using enhancer reporter lines in WT, E(z)-KD and CBP-KD for *Doc2* and *Ptx1.* White arrows indicate aberrant expression patterns of the reporter upon E(z) depletion. A, anterior; P, posterior; D, dorsal; V, ventral.

c, Top, UMAP embedding of our projected cells onto the sci-RNA-seq or sci-ATAC-seq datasets[56]. Cells are categorized based on the projected timing (early, middle or late), corresponding to their different time collections and recapitulating the GEX and ATAC dynamics during early embryogenesis.

d, Relative fractions of cells within 10x Multiome WT, E(z)-KD and CBP-KD timing projection for both scRNA-seq and scATAC-seq.

e, UMAP embedding and bar plot of the projected scRNA-seq (GEX) data colored by the annotated germ layers in each developmental time point.

f, g, UMAP embedding of ForceAtlas2 (FA) pseudotime coloured by WT, E(z)-KD and CBP-KD conditions, showing the transcriptional delay of CBP and the specification timing of the different germ layers.

h, Differentiation scheme of the ZGA embryo based on the pseudotime analysis. Yolk cells are the first to be specified, while neural ectoderm and mesoderm cells are the latest in the differentiation trajectory.

Supplementary Figure 8

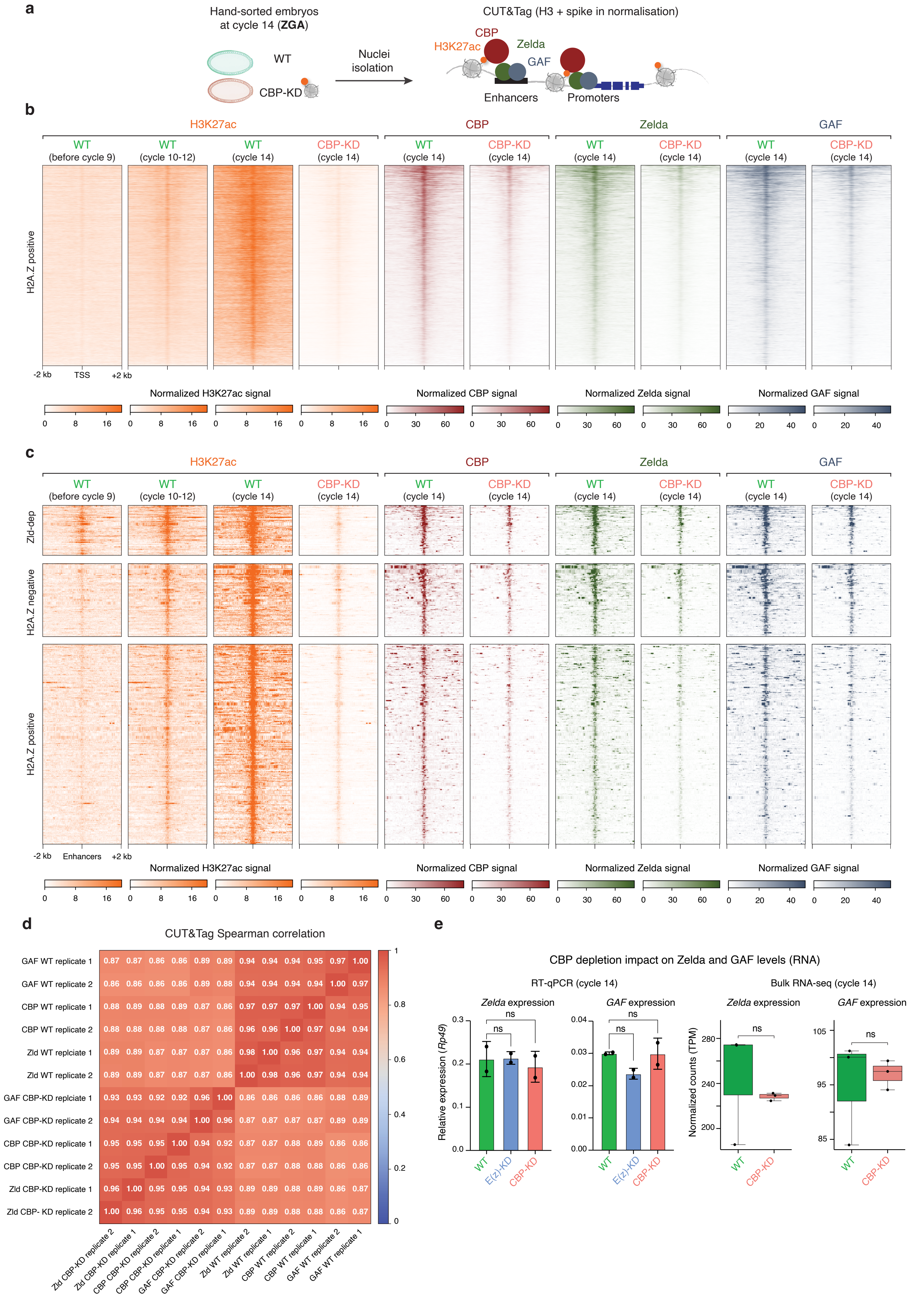

Supplementary Fig. 8: CBP depletion impairs pioneer factors binding at both promoters and enhancers of zygotically active genes.

a, Experimental design. Wild-type (WT) and CBP-KD embryos are precisely hand-selected at cycle 14. Nuclei are isolated and processed for CUT&Tag. Each experiment is normalized to its H3 CUT&Tag as "input" control and to library spike-in (see Methods).

b, Heat map of H2A.Z positive active genes (GRO-seq) at ZGA[23]. Signal is centred around +/- 2kb from TSS. Sorting is based on H3K27ac levels before cycle 9 and shows CUT&Tag signal distribution of this mark at three developmental time points and signal distribution of CBP, Zelda and GAF at ZGA in both wild-type and CBP-KD at cycle 14 (ZGA).

c, Heat map of active genes (GRO-seq) at ZGA clustered based on their dependency on Zelda (zld-dep), H2A.Z- (H2A.Z negative) and H2A.Z+ (H2A.Z positive)[23]. Signal is centred on associated enhancers around +/- 10kb from promoters. Sorting is based on H3K27ac levels before cycle 9 and shows CUT&Tag signal distribution of this mark at three developmental time points and signal distribution of CBP, Zelda and GAF at ZGA in both wild-type and CBP-KD at cycle 14 (ZGA).

d, Spearman correlation coefficient heat maps across biological replicates and conditions of the different CUT&Tags for CBP, Zelda and GAF.

e, *Zelda* and *GAF* mRNA levels in wild-type CBP-KD embryos at cycle 14, measured by RT-qPCR and bulk RNA-seq. Each dot represents a biological replicate. Two-sided Mann–Whitney test. For RT-qPCR, data are presented as mean values ± SD. For total RNA-seq, the lower and upper quartiles of the boxes are showed (Q1 and Q3, respectively). Whiskers, 1.5 × IQR below Q1 and above Q3. Source data are provided as Source Data file.

Supplementary Figure 9

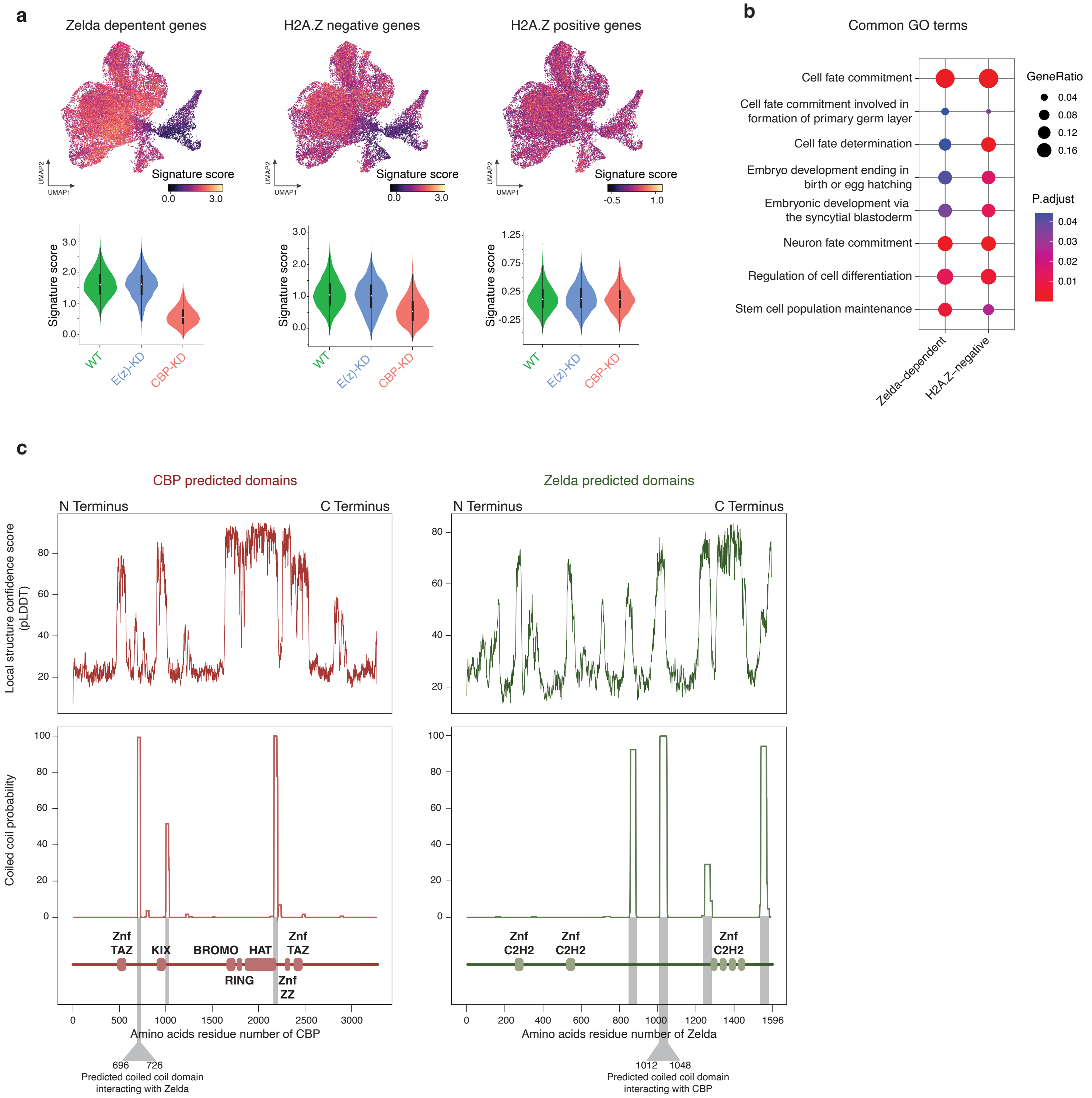

Supplementary Fig. 9: Cell fate related genes are downregulated upon CBP depletion.
a, Top, Gene signature score of Zelda dependent, H2A.Z negative or H2A.Z positive genes in the UMAP embedding of the integrated scRNA-seq of wild-type, E(z)-KD and CBP-KD datasets. Bottom, Violin plot quantification of signature score enrichment across conditions.
b, Gene ontology of commonly enriched terms in Zelda dependent and H2A.Z negative genes. Both are enriched in cell fate related processes. Source data are provided as Source Data file.
c, Top, Local structure confidence score (pLDDT) predicted by Alphafold3 for CBP and Zelda. Bottom, probability that residues of CBP or Zelda make a coiled-coil structure. A schematic of CBP and Zelda structures is provided with the respective amino acid numbers. Gray boxes indicate potential coiled coil regions, the gray triangle indicates the stretches of amino acids which are predicted to interact between CBP and Zelda.

Supplementary Figure 10

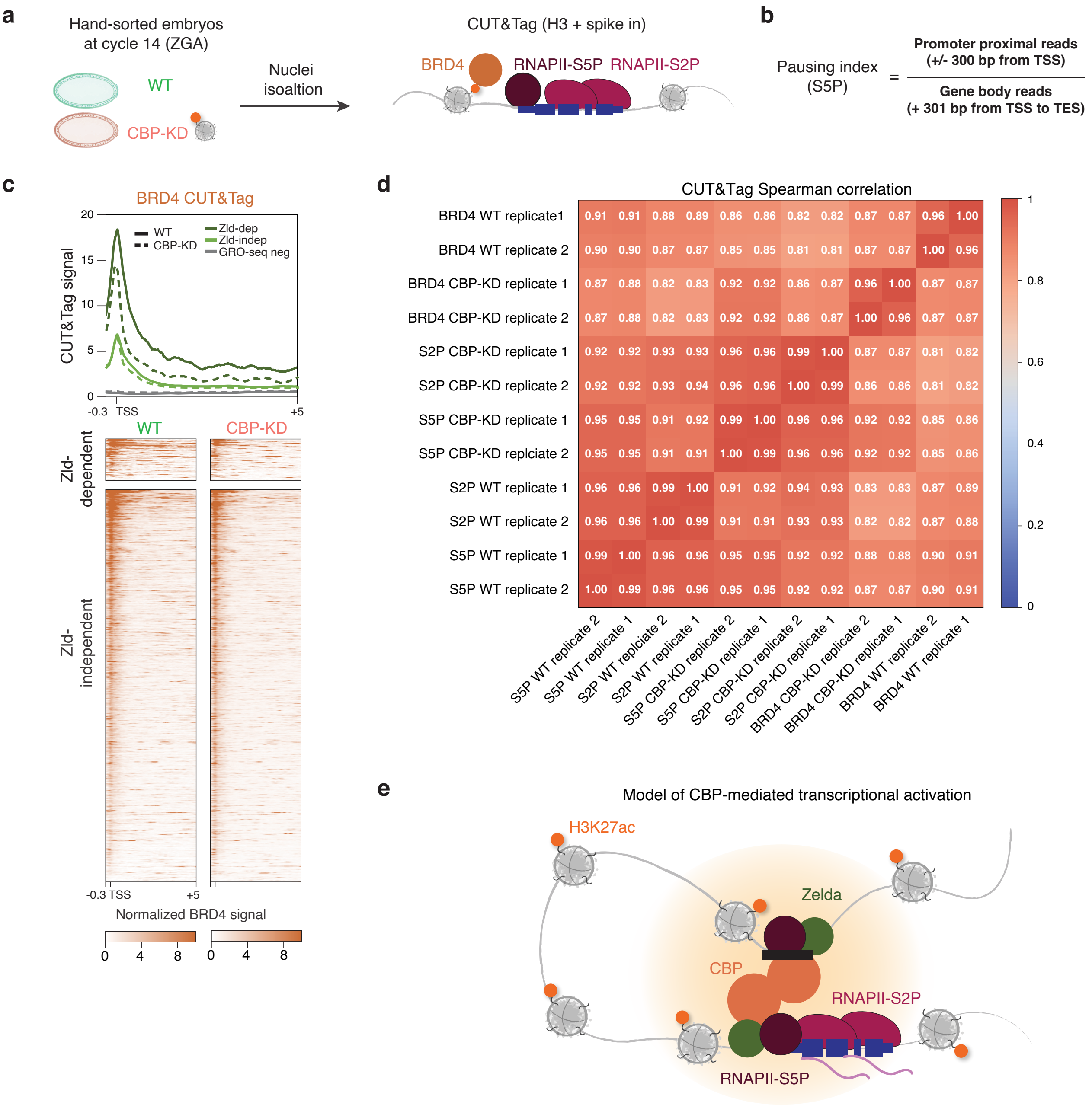

Supplementary Fig. 10: Loss of productive elongation at ZGA is associated with reduced BRD4 binding in CBP-KD embryos.

a, Experimental design. Wild-type (WT) and CBP-KD embryos are precisely hand-selected at cycle 14. Nuclei are isolated and processed for CUT&Tag. Each experiment is normalized to its H3 CUT&Tag as "input" control and to library spike-in (see Methods).

b, Schematic of the pausing index score calculation. Pausing index is computed by doing the ratio of RNAPII-S5P CUT&Tag signal distribution at promoters (+/- 300 bp from TSS) and gene body (+ 301 bp from TSS until TES).

c, Heat map of active genes (GRO-seq) at ZGA clustered based on their dependency on Zelda (zld-dep) or independency (zld-independent)[23]. Signal is centred on gene bodies around - 300 bp and + 5kb from TSS. Sorting is based on GRO-seq levels at cycle 14 and shows CUT&Tag signal distribution of BRD4 in both wild-type and CBP-KD at cycle 14 (ZGA).

d, Spearman correlation coefficient heat maps across biological replicates and conditions of the different CUT&Tags for RNAPII-S5P, RNAPII-S2P and BRD4.

e, Model of CBP's role in pioneer factor binding stabilization and of RNAPII release in productive elongation during ZGA. Gray line schetches the chromatin fiber. H3K27ac is represented by orange dots. Gene region is represented by blue boxes. Enhancer region is represented by a black box. CBP is coloured in orange, Zelda in green, RNA Polymerase II serine 5 phosphorilation (RNAPII-S5P) in dark red, RNA Polymerase II serine 2 phosphorilation (RNAPII-S2P) in purple and nascent mRNA in pink.

Supplementary Data legends:

Supplementary Data 1. Set of H3K27me3, H3K27ac or H3K27me3/ac peaks at three developmental stages. List of CUT&Tag peaks used in this study across three developmental stages and their genomic classification, related to Figure 1 and Supplementary Figure 1 and 2.

Supplementary Data 2. Germ layers marker genes. List of marker genes used to annotate the germ layers precursors in this study.

Supplementary Data 3. Highly variable genes or marker genes and their most accessible promoter and highest score linked peak. List of highly variable genes or germ layer marker genes used in this study with their relative promoter or enhancer at ZGA, related to Figure 2 and Supplementary Figure 3.

Supplementary Data 4. Highly expressed genes in annotated germ layers from the integrated 10x Multiome. List of genes that are highly expressed per germ layer across the WT, E(z)-KD and CBP-KD scRNA-seq integration, related to Figure 5. Wilcoxon rank-sum two-sided, corrected with Benjamin-Hochberg.

Supplementary Data 5. Percentage of cells within each germ layer from 10x Multiome in WT, E(z)-KD and CBP-KD. List of raw or normalized number of cells within each germ layer across different condition, related to Figure 3 and Supplementary Figure 5.

Supplementary Data 6. Differential gene expression and differential accessibility between E(z)-KD or CBP-KD and WT of promoter or enhancer regions across germ layers from 10X Multiome. Differential gene expression or differential accessibility (at promoters or enhancers) of marker genes upon E(z) or CBP depletion, related to Supplementary Figure 5. Wilcoxon rank-sum two-sided, corrected with Benjamin-Hochberg.

Supplementary Data 7. Fly lines. Description of all fly lines used in this study.

Supplementary Data 8. Antibodies. Description of all antibodies used in this study.

Supplementary Data 9. 10x Multiome scATAC-seq thresholds. List of scATAC-seq minimum quality thresholds used in this study.
