# Supplementary material for: Chromatin landscape at cis-regulatory elements orchestrates cell fate decisions in early embryogenesis
Source: Nat Commun. 2025 Mar 27;16:3007. doi: 10.1038/s41467-025-57719-4 (PMC11950382; doi:10.1038/s41467-025-57719-4)
Supplement: Supplementary file 2 — Description of Additional Supplementary Files [file 41467_2025_57719_MOESM2_ESM.pdf]

Description of Additional Supplementary Files

Supplementary Data 1. Set of H3K27me3, H3K27ac or H3K27me3/ac peaks at three developmental stages. List of CUT&Tag peaks used in this study across three developmental stages and their genomic classification, related to Figure 1 and Supplementary Figure 1 and 2.

Supplementary Data 2. Germ layers marker genes. List of marker genes used to annotate the germ layers precursors in this study.

Supplementary Data 3. Highly variable genes or marker genes and their most accessible promoter and highest score linked peak. List of highly variable genes or germ layer marker genes used in this study with their relative promoter or enhancer at ZGA, related to Figure 2 and Supplementary Figure 3.

Supplementary Data 4. Highly expressed genes in annotated germ layers from the integrated 10x Multiome. List of genes that are highly expressed per germ layer across the WT, E(z)-KD and CBP-KD scRNA-seq integration, related to Figure 5. Wilcoxon rank-sum two-sided, corrected with Benjamin-Hochberg.

Supplementary Data 5. Percentage of cells within each germ layer from 10x Multiome in WT, E(z)-KD and CBP-KD. List of raw or normalized number of cells within each germ layer across different condition, related to Figure 3 and Supplementary Figure 5.

Supplementary Data 6. Differential gene expression and differential accessibility between E(z)-KD or CBP-KD and WT of promoter or enhancer regions across germ layers from 10X Multiome. Differential gene expression or differential accessibility (at promoters or enhancers) of marker genes upon E(z) or CBP depletion, related to Supplementary Figure 5. Wilcoxon rank-sum two-sided, corrected with Benjamin-Hochberg.

Supplementary Data 7. Fly lines. Description of all fly lines used in this study.

Supplementary Data 8. Antibodies. Description of all antibodies used in this study.

Supplementary Data 9. 10x Multiome scATAC-seq thresholds. List of scATAC-seq minimum quality thresholds used in this study.
