# Supplementary material for: Chromatin landscape at cis-regulatory elements orchestrates cell fate decisions in early embryogenesis
Source: Nat Commun. 2025 Mar 27;16:3007. doi: 10.1038/s41467-025-57719-4 (PMC11950382; doi:10.1038/s41467-025-57719-4)
Supplement: Supplementary file 12 — Reporting Summary [file 41467_2025_57719_MOESM12_ESM.pdf]

nature portfolio

Corresponding author(s): Nicola Iovino

Last updated by author(s): Feb, 2025

Reporting Summary

Nature Portfolio wishes to improve the reproducibility of the work that we publish. This form provides structure for consistency and transparency in reporting. For further information on Nature Portfolio policies, see our Editorial Policies and the Editorial Policy Checklist.

Statistics

For all statistical analyses, confirm that the following items are present in the figure legend, table legend, main text, or Methods section.

n/a	Confirmed	
☐	☒	The exact sample size (*n*) for each experimental group/condition, given as a discrete number and unit of measurement
☐	☒	A statement on whether measurements were taken from distinct samples or whether the same sample was measured repeatedly
☐	☒	The statistical test(s) used AND whether they are one- or two-sided<br>*Only common tests should be described solely by name; describe more complex techniques in the Methods section.*
☐	☒	A description of all covariates tested
☐	☒	A description of any assumptions or corrections, such as tests of normality and adjustment for multiple comparisons
☐	☒	A full description of the statistical parameters including central tendency (e.g. means) or other basic estimates (e.g. regression coefficient) AND variation (e.g. standard deviation) or associated estimates of uncertainty (e.g. confidence intervals)
☐	☒	For null hypothesis testing, the test statistic (e.g. *F*, *t*, *r*) with confidence intervals, effect sizes, degrees of freedom and *P* value noted<br>*Give P values as exact values whenever suitable.*
☐	☒	For Bayesian analysis, information on the choice of priors and Markov chain Monte Carlo settings
☒	☐	For hierarchical and complex designs, identification of the appropriate level for tests and full reporting of outcomes
☐	☒	Estimates of effect sizes (e.g. Cohen's *d*, Pearson's *r*), indicating how they were calculated

Our web collection on statistics for biologists contains articles on many of the points above.

Software and code

Policy information about availability of computer code

Data collection	Zen (Zeiss) was used to record immunofluorescence the data.
Data analysis	The code for the analysis is deposited in a Github repository (https://github.com/dimadatascience/scmultiome).<br>Further details are available in the Methods.

For manuscripts utilizing custom algorithms or software that are central to the research but not yet described in published literature, software must be made available to editors and reviewers. We strongly encourage code deposition in a community repository (e.g. GitHub). See the Nature Portfolio guidelines for submitting code & software for further information.

Data

Policy information about availability of data

All manuscripts must include a data availability statement. This statement should provide the following information, where applicable:

- Accession codes, unique identifiers, or web links for publicly available datasets
- A description of any restrictions on data availability
- For clinical datasets or third party data, please ensure that the statement adheres to our policy

All ChIP-seq, CUT&Tag, ATAC-seq, RNA-seq and 10x Multiome data generated in this study are deposited in GEO under the accession numbers:
GSE269361 (https://www.ncbi.nlm.nih.gov/geo/query/acc.cgi?acc=GSE269361),
GSE269362 (https://www.ncbi.nlm.nih.gov/geo/query/acc.cgi?acc=GSE269362),

GSE269365 (https://www.ncbi.nlm.nih.gov/geo/query/acc.cgi?acc=GSE269365),
GSE269367 (https://www.ncbi.nlm.nih.gov/geo/query/acc.cgi?acc=GSE269367),
GSE269368 (https://www.ncbi.nlm.nih.gov/geo/query/acc.cgi?acc=GSE269368),
GSE289288 (https://www.ncbi.nlm.nih.gov/geo/query/acc.cgi?acc=GSE289288) and
GSE289289 ((https://www.ncbi.nlm.nih.gov/geo/query/acc.cgi?acc=GSE289289).

GSM8313859 WT, mesoderm, input, repl1
GSM8313860 WT, mesoderm, H27me3, repl1
GSM8313861 WT, mesoderm, input, repl2
GSM8313862 WT, mesoderm, H27me3, repl2
GSM8313908 CUTTAG, H3, WT, c14, repl1
GSM8313909 CUTTAG, H3K27me3, WT, c14, repl1
GSM8313910 CUTTAG, H3K27ac, WT, c14, repl1
GSM8313911 CUTTAG, H3, WT, c14, repl2
GSM8313912 CUTTAG, H3K27me3, c14, repl2
GSM8313913 CUTTAG, H3K27ac, WT, c14, repl2
GSM8313914 CUTTAG, H3,CBPKD, c14, repl1
GSM8313915 CUTTAG, H3K27ac, CBPKD, c14, repl1
GSM8313916 CUTTAG, H3, EZKD, c14, repl1
GSM8313917 CUTTAG, H3K27me3, EZKD, c14, repl1
GSM8313918 CUTTAG, H3K27ac, EZKD, c14, rep1
GSM8313919 CUTTAG, H3, CBPKD, c14, repl2
GSM8313920 CUTTAG, H3K27ac, CBPKD, c14, repl2
GSM8313921 CUTTAG, H3, EZKD, c14, repl2
GSM8313922 CUTTAG, H3K27me3, EZKD, c14, repl2
GSM8313923 CUTTAG, H3K27ac, EZKD, c14, repl2
GSM8313924 CUTTAG, H3, WT, bfc9, repl1
GSM8313925 CUTTAG, H3K27me3, WT, bfc9, repl1
GSM8313926 CUTTAG, H3K27ac, WT, bfc9, repl1
GSM8313927 CUTTAG, H3, WT, bfc9, repl2
GSM8313928 CUTTAG, H3K27me3, WT, bfc9, repl2
GSM8313929 CUTTAG, H3K27ac, WT, bfc9, repl2
GSM8313930 CUTTAG, H3, WT, c10-12, repl1
GSM8313931 CUTTAG, H3K27me3, WT, c10-12, repl1
GSM8313932 CUTTAG, H3K27ac, WT, c10-12, repl1
GSM8313933 CUTTAG, H3, WT, c10-12, repl2
GSM8313934 CUTTAG, H3K27me3, WT, c10-12, repl2
GSM8313935 CUTTAG, H3K27ac, WT, c10-12, repl2
GSM8313936 CUTTAG, H3 ATAC-seq normalization, WT, st3, repl1
GSM8313937 CUTTAG, H3 ATAC-seq normalization, WT, st4, repl1
GSM8313938 CUTTAG, H3 ATAC-seq normalization, WT, st5, repl1
GSM8313940 CUTTAG, H3 ATAC-seq normalization, WT, st3, repl2
GSM8313942 CUTTAG, H3 ATAC-seq normalization, WT, st4, repl2
GSM8313944 CUTTAG, H3 ATAC-seq normalization, WT, st5, repl2
GSM8313946 CUTTAG, H3 cofactors normalization, WT, c14, repl1
GSM8313948 CUTTAG, CBP, WT, c14, repl1
GSM8313950 CUTTAG, Zld, WT, c14, repl1
GSM8313952 CUTTAG, GAF, WT, c14, repl1
GSM8313954 CUTTAG, H3 cofactors normalization, WT, c14, repl2
GSM8313955 CUTTAG, CBP, WT, c14, repl2
GSM8313956 CUTTAG, Zld, WT, c14, repl2
GSM8313957 CUTTAG, GAF, WT, c14, repl2
GSM8313958 CUTTAG, H3 cofactors normalization, EzKD, c14, repl1
GSM8313959 CUTTAG, CBP, EzKD, c14, repl1
GSM8313960 CUTTAG, H3 cofactors normalization, E-KD, c14, repl2
GSM8313961 CUTTAG, CBP, EzKD, c14, repl2
GSM8313962 CUTTAG, H3 cofactors normalization, CBPKD, c14, repl1
GSM8313963 CUTTAG, CBP, CBPKD, c14, repl1
GSM8313964 CUTTAG, Zld, CBPKD, c14, repl1
GSM8313965 CUTTAG, GAF, CBPKD, c14, repl1
GSM8313966 CUTTAG, H3 cofactors normalization, CBPKD, c14, repl2
GSM8313967 CUTTAG, CBP, CBPKD, c14, repl2
GSM8313968 CUTTAG, Zld, CBPKD, c14, repl2
GSM8313969 CUTTAG, GAF, CBPKD, c14, repl2
GSM8313899 bulkRNAseq, WT, c14, repl1
GSM8313900 bulkRNAseq, WT, c14, repl2
GSM8313901 bulkRNAseq, WT, c14, repl3
GSM8313902 bulkRNAseq, EzKD, c14, repl1

GSM8313903 bulkRNAseq, EzKD, c14, repl2
GSM8313904 bulkRNAseq, EzKD, c14, repl3
GSM8313905 bulkRNAseq, CBPKD, c14, repl1
GSM8313906 bulkRNAseq, CBPKD, c14, repl2
GSM8313907 bulkRNAseq, CBPKD, c14, repl3
GSM8313863 ATAC-seq, WT, bfc9, repl1
GSM8313864 ATAC-seq, WT, c10-12, repl1
GSM8313865 ATAC-seq, WT, c14, repl1
GSM8313866 ATAC-seq, WT, bfc9, repl2
GSM8313867 ATAC-seq, WT, c10-12, repl2
GSM8313868 ATAC-seq, WT, c14, repl2
GSM8313970 Multiome, scRNAseq, WT, c14, rep1
GSM8313971 Multiome, scRNAseq, WT, c14, rep2
GSM8313972 Multiome, scRNAseq, CBPKD, c14, rep1
GSM8313973 Multiome, scRNAseq, CBPKD, c14, rep2
GSM8313974 Multiome, scRNAseq, EzKD, c14, rep1
GSM8313975 Multiome, scRNAseq, EzKD, c14, rep2
GSM8313976 Multiome, scATACseq, WT, c14, rep1
GSM8313977 Multiome, scATACseq, WT, c14, rep2
GSM8313978 Multiome, scATACseq, CBPKD, c14, rep1
GSM8313979 Multiome, scATACseq, CBPKD, c14, rep2
GSM8313980 Multiome, scATACseq, EzKD, c14, rep1
GSM8313981 Multiome, scATACseq, EzKD, c14, rep2
GSM8788433 total, bulkRNAseq, WT, bfc9, repl1
GSM8788434 total, bulkRNAseq, WT, bfc9, repl2
GSM8788435 total, bulkRNAseq, WT, bfc9, repl3
GSM8788436 total, bulkRNAseq, CBP-KD, bfc9, repl1
GSM8788437 total, bulkRNAseq, CBP-KD, bfc9, repl2
GSM8788438 total, bulkRNAseq, CBP-KD, bfc9, repl3
GSM8788439 total, bulkRNAseq, E(z)-KD, bfc9, repl1
GSM8788440 total, bulkRNAseq, E(z)-KD, bfc9, repl2
GSM8788441 total, bulkRNAseq, E(z)-KD, bfc9, repl3
GSM8788442 total, bulkRNAseq, WT, c14, repl1
GSM8788443 total, bulkRNAseq, WT, c14, repl2
GSM8788444 total, bulkRNAseq, WT, c14, repl3
GSM8788445 total, bulkRNAseq, CBP-KD, c14, repl1
GSM8788446 total, bulkRNAseq, CBP-KD, c14, repl2
GSM8788447 total, bulkRNAseq, CBP-KD, c14, repl3
GSM8788448 total, bulkRNAseq, E(z)-KD, c14, repl1
GSM8788449 total, bulkRNAseq, E(z)-KD, c14, repl2
GSM8788450 total, bulkRNAseq, E(z)-KD, c14, repl3
GSM8788451 CUTTAG, H3 RNAPII normalization, WT, c14, repl1
GSM8788452 CUTTAG, H3 RNAPII normalization, WT, c14, repl2
GSM8788453 CUTTAG, RNAPII-S2P, WT, c14, repl1
GSM8788454 CUTTAG, RNAPII-S2P, WT, c14, repl2
GSM8788455 CUTTAG, RNAPII-S5P, WT, c14, repl1
GSM8788456 CUTTAG, RNAPII-S5P, WT, c14, repl2
GSM8788457 CUTTAG, H3 RNAPII normalization, CBPKD, c14, repl1
GSM8788458 CUTTAG, H3 RNAPII normalization, CBPKD, c14, repl2
GSM8788459 CUTTAG, RNAPII-S2P, CBPKD, c14, repl1
GSM8788460 CUTTAG, RNAPII-S2P, CBPKD, c14, repl2
GSM8788461 CUTTAG, RNAPII-S5P, CBPKD, c14, repl1
GSM8788462 CUTTAG, RNAPII-S5P, CBPKD, c14, repl2
GSM8788463 CUTTAG, BRD4, WT, c14, repl1
GSM8788464 CUTTAG, BRD4, WT, c14, repl2
GSM8788465 CUTTAG, BRD4, CBPKD, c14, repl1
GSM8788466 CUTTAG, BRD4, CBPKD, c14, repl2

We downloaded the H3K27ac and Pol II ChIP-seq in mesoderm sorted nuclei from the ENA portal (https://www.ebi.ac.uk/ena) with identifier ERP000560.

Research involving human participants, their data, or biological material

Policy information about studies with human participants or human data. See also policy information about sex, gender (identity/presentation), and sexual orientation and race, ethnicity and racism.

Reporting on sex and gender

Use the terms sex (biological attribute) and gender (shaped by social and cultural circumstances) carefully in order to avoid confusing both terms. Indicate if findings apply to only one sex or gender; describe whether sex and gender were considered in study design; whether sex and/or gender was determined based on self-reporting or assigned and methods used.

	Provide in the source data disaggregated sex and gender data, where this information has been collected, and if consent has been obtained for sharing of individual-level data; provide overall numbers in this Reporting Summary. Please state if this information has not been collected. *Report sex- and gender-based analyses where performed, justify reasons for lack of sex- and gender-based analysis.*
Reporting on race, ethnicity, or other socially relevant groupings	*Please specify the socially constructed or socially relevant categorization variable(s) used in your manuscript and explain why they were used. Please note that such variables should not be used as proxies for other socially constructed/relevant variables (for example, race or ethnicity should not be used as a proxy for socioeconomic status).* *Provide clear definitions of the relevant terms used, how they were provided (by the participants/respondents, the researchers, or third parties), and the method(s) used to classify people into the different categories (e.g. self-report, census or administrative data, social media data, etc.)* *Please provide details about how you controlled for confounding variables in your analyses.*
Population characteristics	*Describe the covariate-relevant population characteristics of the human research participants (e.g. age, genotypic information, past and current diagnosis and treatment categories). If you filled out the behavioural & social sciences study design questions and have nothing to add here, write "See above."*
Recruitment	*Describe how participants were recruited. Outline any potential self-selection bias or other biases that may be present and how these are likely to impact results.*
Ethics oversight	*Identify the organization(s) that approved the study protocol.*

Note that full information on the approval of the study protocol must also be provided in the manuscript.

Field-specific reporting

Please select the one below that is the best fit for your research. If you are not sure, read the appropriate sections before making your selection.

☒ Life sciences ☐ Behavioural & social sciences ☐ Ecological, evolutionary & environmental sciences

For a reference copy of the document with all sections, see nature.com/documents/nr-reporting-summary-flat.pdf

Life sciences study design

All studies must disclose on these points even when the disclosure is negative.

Sample size	10x Multiome experiments have been performed in biological replicates. For bulk RNA-seq 3 biological replicates were collected. All CUT&Tag, ChIP-seq and bulk ATAC-seq experiments have been performed in biological replicates. We did not apply any statistical methods to pre-determine sample size and followed the general standard practice in the field. The number of replicates in experiments is also stated in the legends.
Data exclusions	We did not exclude data.
Replication	We performed all experiments in biological replicates and could observe agreement between the replicates. 10x Multiome experiments were performed with 2 biologically independent samples for each condition. RT-qPCR, CUT&Tag, ChIP-seq and ATAC-seq were performed with 2 biologically independent samples for each condition or stage of development. Bulk RNA-seq experiments were performed with 3 biologically independent samples for each condition.
Randomization	No experiments that required randomization of the samples were performed. We controlled variability by collecting biologically independent samples in several batches. Embryos from the same developmental stage or cycle were collected in pools.
Blinding	No experiments that required binding of groups were performed. Binding was not possible given the noticeable differences in phenotype between the mutant embryos and the controls.

Reporting for specific materials, systems and methods

We require information from authors about some types of materials, experimental systems and methods used in many studies. Here, indicate whether each material, system or method listed is relevant to your study. If you are not sure if a list item applies to your research, read the appropriate section before selecting a response.

Materials & experimental systems

n/a	Involved in the study	
	x	Antibodies
x		Eukaryotic cell lines
x		Palaeontology and archaeology
	x	Animals and other organisms
x		Clinical data
x		Dual use research of concern
x		Plants

Methods

n/a	Involved in the study	
	x	ChIP-seq
x		Flow cytometry
x		MRI-based neuroimaging

Antibodies

Antibodies used

A description of the antibodies used in this study is available in Supplementary Table 8.
H3K27me3 Diagenode C15410195
H3K27ac Diagenode C15410196
H3K27me3 Thomas Jenuwein Lab
H3K27ac Wako 306-34849
H3 Active Motif 39763
CBP Yuri Schwartz Lab
Zelda Melissa Harrison Lab
GAF Maxim Erokhin and Daria Chetverina Labs
RNAPII-S5P Abcam, ab5131
RNAPII-S2P Abcam, ab5095
BRD4 Renato Paro Lab
Rabbit anti mouse Abcam ab46540
guinea pig anti rabbit antibodies online ABIN101961
Rabbit IgG (H+L) Molecular Probes by ThermoScientific A11070
mouse IgG (H+L) Molecular Probes by ThermoScientific A21425

Validation

The antibodies used in this study are commercially available and have been validated by manufacturer. We have further validated the antibodies against H3K27me3, H3K27ac, CBP, Zelda, GAF either by Immunofluorescence Staining, Western blot or CUT&Tag in the control and the knockdown of the respective epigenetic writer or the protein itself.

Animals and other research organisms

Policy information about studies involving animals; ARRIVE guidelines recommended for reporting animal research, and Sex and Gender in Research

Laboratory animals

We used Drosophila melanogaster embryos to perform all experiments in this study.
A detailed list of the fly lines used in this study is available in Supplementary Table 7.
shRNA E(z) BDSC #33695
WT BDSC#36303
shRNA CBP Iovino Lab
matα4-GAL-VP16 BDSC #7063
w*;pDest-BDP-LacZw_VT27867 (Doc2) Iovino Lab
w* ;pDest-BDP-LacZw_VT27870-(Doc2) Iovino Lab
w*pDest-BDP-LacZw_VT50187-(Ptx1) Iovino Lab
w* pDest-BDP-LacZw_VT50192-(Ptx-1) Iovino Lab
w*;pDest-BDP-LacZw_VT27867-(Doc2); Valium 20 shRNA E(z) Iovino Lab
w*;pDest-BDP-LacZw_VT27870-(Doc2) ; Valium 20 shRNA E(z) Iovino Lab
w*; pDest-BDP-LacZw_VT50192-(Ptx1); Valium 20 shRNA E(z) Iovino Lab
w*;pDest-BDP-LacZw_VT50187-(Ptx1); Valium 20 shRNA E(z) Iovino Lab
w*;pDest-BDP-LacZw_VT27867-(Doc2); Walium20 shRNA nej-3UTR-1 Iovino Lab
w*;pDest-BDP-LacZw_VT27870-(Doc2); Walium20 shRNA nej-3UTR-1 Iovino Lab
w*;pDest-BDP-LacZw_VT50192-(Ptx1); Walium20 shRNA nej-3UTR-1 Iovino Lab
w*;pDest-BDP-LacZw_VT50187-(Ptx1); Walium20 shRNA nej-3UTR-1 Iovino Lab

Wild animals

This study did not involve the use of wild animals.

Reporting on sex

Indicate if findings apply to only one sex; describe whether sex was considered in study design, methods used for assigning sex. Provide data disaggregated for sex where this information has been collected in the source data as appropriate; provide overall numbers in this Reporting Summary. Please state if this information has not been collected. Report sex-based analyses where performed, justify reasons for lack of sex-based analysis.

Field-collected samples	This study did not involve samples collected from the field.
Ethics oversight	All work was conducted in Drosophila melanogaster, an invertebrate animal from the insect group. Invertebrate models are not regulated by the TierSchVersV and therefore are not subjected to ethical approval.

Note that full information on the approval of the study protocol must also be provided in the manuscript.

Plants

Seed stocks	*Report on the source of all seed stocks or other plant material used. If applicable, state the seed stock centre and catalogue number. If plant specimens were collected from the field, describe the collection location, date and sampling procedures.*
Novel plant genotypes	*Describe the methods by which all novel plant genotypes were produced. This includes those generated by transgenic approaches, gene editing, chemical/radiation-based mutagenesis and hybridization. For transgenic lines, describe the transformation method, the number of independent lines analyzed and the generation upon which experiments were performed. For gene-edited lines, describe the editor used, the endogenous sequence targeted for editing, the targeting guide RNA sequence (if applicable) and how the editor was applied.*
Authentication	*Describe any authentication procedures for each seed stock used or novel genotype generated. Describe any experiments used to assess the effect of a mutation and, where applicable, how potential secondary effects (e.g. second site T-DNA insertions, mosiacism, off-target gene editing) were examined.*

ChIP-seq

Data deposition

☒ Confirm that both raw and final processed data have been deposited in a public database such as GEO.

☒ Confirm that you have deposited or provided access to graph files (e.g. BED files) for the called peaks.

Data access links<br>*May remain private before publication.*	GEO accession GSE269361: https://www.ncbi.nlm.nih.gov/geo/query/acc.cgi?acc=GSE269361<br>GEO accession GSE269367: https://www.ncbi.nlm.nih.gov/geo/query/acc.cgi?acc=GSE269367<br>GEO accession GSE289289 https://www.ncbi.nlm.nih.gov/geo/query/acc.cgi?acc=GSE289289
Files in database submission	GSM8313859 WT, mesoderm, input, repl1<br>GSM8313860 WT, mesoderm, H27me3, repl1<br>GSM8313861 WT, mesoderm, input, repl2<br>GSM8313862 WT, mesoderm, H27me3, repl2<br>GSM8313908 CUTTAG, H3, WT, c14, repl1<br>GSM8313909 CUTTAG, H3K27me3, WT, c14, repl1<br>GSM8313910 CUTTAG, H3K27ac, WT, c14, repl1<br>GSM8313911 CUTTAG, H3, WT, c14, repl2<br>GSM8313912 CUTTAG, H3K27me3, c14, repl2<br>GSM8313913 CUTTAG, H3K27ac, WT, c14, repl2<br>GSM8313914 CUTTAG, H3,CBPKD, c14, repl1<br>GSM8313915 CUTTAG, H3K27ac, CBPKD, c14, repl1<br>GSM8313916 CUTTAG, H3, EZKD, c14, repl1<br>GSM8313917 CUTTAG, H3K27me3, EZKD, c14, repl1<br>GSM8313918 CUTTAG, H3K27ac, EZKD, c14, rep1<br>GSM8313919 CUTTAG, H3, CBPKD, c14, repl2<br>GSM8313920 CUTTAG, H3K27ac, CBPKD, c14, repl2<br>GSM8313921 CUTTAG, H3, EZKD, c14, repl2<br>GSM8313922 CUTTAG, H3K27me3, EZKD, c14, repl2<br>GSM8313923 CUTTAG, H3K27ac, EZKD, c14, repl2<br>GSM8313924 CUTTAG, H3, WT, bfc9, repl1<br>GSM8313925 CUTTAG, H3K27me3, WT, bfc9, repl1<br>GSM8313926 CUTTAG, H3K27ac, WT, bfc9, repl1<br>GSM8313927 CUTTAG, H3, WT, bfc9, repl2<br>GSM8313928 CUTTAG, H3K27me3, WT, bfc9, repl2<br>GSM8313929 CUTTAG, H3K27ac, WT, bfc9, repl2<br>GSM8313930 CUTTAG, H3, WT, c10-12, repl1<br>GSM8313931 CUTTAG, H3K27me3, WT, c10-12, repl1<br>GSM8313932 CUTTAG, H3K27ac, WT, c10-12, repl1<br>GSM8313933 CUTTAG, H3, WT, c10-12, repl2<br>GSM8313934 CUTTAG, H3K27me3, WT, c10-12, repl2<br>GSM8313935 CUTTAG, H3K27ac, WT, c10-12, repl2<br>GSM8313936 CUTTAG, H3 ATAC-seq normalization, WT, st3, repl1<br>GSM8313937 CUTTAG, H3 ATAC-seq normalization, WT, st4, repl1

GSM8313938 CUTTAG, H3 ATAC-seq normalization, WT, st5, repl1
GSM8313940 CUTTAG, H3 ATAC-seq normalization, WT, st3, repl2
GSM8313942 CUTTAG, H3 ATAC-seq normalization, WT, st4, repl2
GSM8313944 CUTTAG, H3 ATAC-seq normalization, WT, st5, repl2
GSM8313946 CUTTAG, H3 cofactors normalization, WT, c14, repl1
GSM8313948 CUTTAG, CBP, WT, c14, repl1
GSM8313950 CUTTAG, Zld, WT, c14, repl1
GSM8313952 CUTTAG, GAF, WT, c14, repl1
GSM8313954 CUTTAG, H3 cofactors normalization, WT, c14, repl2
GSM8313955 CUTTAG, CBP, WT, c14, repl2
GSM8313956 CUTTAG, Zld, WT, c14, repl2
GSM8313957 CUTTAG, GAF, WT, c14, repl2
GSM8313958 CUTTAG, H3 cofactors normalization, EzKD, c14, repl1
GSM8313959 CUTTAG, CBP, EzKD, c14, repl1
GSM8313960 CUTTAG, H3 cofactors normalization, E-KD, c14, repl2
GSM8313961 CUTTAG, CBP, EzKD, c14, repl2
GSM8313962 CUTTAG, H3 cofactors normalization, CBPKD, c14, repl1
GSM8313963 CUTTAG, CBP, CBPKD, c14, repl1
GSM8313964 CUTTAG, Zld, CBPKD, c14, repl1
GSM8313965 CUTTAG, GAF, CBPKD, c14, repl1
GSM8313966 CUTTAG, H3 cofactors normalization, CBPKD, c14, repl2
GSM8313967 CUTTAG, CBP, CBPKD, c14, repl2
GSM8313968 CUTTAG, Zld, CBPKD, c14, repl2
GSM8313969 CUTTAG, GAF, CBPKD, c14, repl2
GSM8788451 CUTTAG, H3 RNAPII normalization, WT, c14, repl1
GSM8788452 CUTTAG, H3 RNAPII normalization, WT, c14, repl2
GSM8788453 CUTTAG, RNAPII-S2P, WT, c14, repl1
GSM8788454 CUTTAG, RNAPII-S2P, WT, c14, repl2
GSM8788455 CUTTAG, RNAPII-S5P, WT, c14, repl1
GSM8788456 CUTTAG, RNAPII-S5P, WT, c14, repl2
GSM8788457 CUTTAG, H3 RNAPII normalization, CBPKD, c14, repl1
GSM8788458 CUTTAG, H3 RNAPII normalization, CBPKD, c14, repl2
GSM8788459 CUTTAG, RNAPII-S2P, CBPKD, c14, repl1
GSM8788460 CUTTAG, RNAPII-S2P, CBPKD, c14, repl2
GSM8788461 CUTTAG, RNAPII-S5P, CBPKD, c14, repl1
GSM8788462 CUTTAG, RNAPII-S5P, CBPKD, c14, repl2
GSM8788463 CUTTAG, BRD4, WT, c14, repl1
GSM8788464 CUTTAG, BRD4, WT, c14, repl2
GSM8788465 CUTTAG, BRD4, CBPKD, c14, repl1
GSM8788466 CUTTAG, BRD4, CBPKD, c14, repl2

Genome browser session
(e.g. UCSC)

Provide a link to an anonymized genome browser session for "Initial submission" and "Revised version" documents only, to enable peer review. Write "no longer applicable" for "Final submission" documents.

Methodology

Replicates

All ChIP-seq or CUT&Tag data were performed in biological replicates.

Sequencing depth

We sequenced all samples in this study at least to a depth of 5 Mio (15 Mio for ChIP-seq).

Antibodies

A detailed list of antibodies used for CUT&Tag is available in Supplementary Table 8.
1 µg of H3K27me3 Diagenode C15410195,
1 µg of H3K27ac Diagenode C15410196
1 µg of H3 Active Motif39763
3 µg of CBP Yuri Schwartz Lab
5 µL Zelda Melissa Harrison Lab
1 µg GAF Maxim Erokhin and Daria Chetverina Labs
1 µg of RNAPII-S5P Abcam, ab5131
1 µg of RNAPII-S2P Abcam, ab5095
3 µL of BRD4 Renato Paro Lab

Peak calling parameters

Macs2 was used to call ChIP-seq or CUT&Tag peaks using the following options: -g dm -q 0.05 –broad. Further details are available in the Methods.

Data quality

We visually inspected all ChIP-seq or CUT&Tag tracks and called peaks in the genome browser. To call the peaks we used a cut-off of 0.05 on the q- value and only included peaks that passed this threshold.

Software

We used snakePipes version 2.4.0 (parameters: --trim --fastqc --properPairs –dedup --mapq 1) with specific CUT&Tag Bowtie2 alignment option (--local --very-sensitive-local --no-discordant --no-mixed -I 10 -X 700). Biological replicates were merged for

downstream analysis. Since the CUT&Tag contains lambda phage spike-ins for reliable quantification of global effects, the libraries were mapped to a constructed hybrid genome of dm6 and lambda phage (NCBI GenBank ID: J02459.1).
